# Effects of Genotype and Growth Temperature on the Contents of Tannin, Phytate and *In Vitro* Iron Availability of Sorghum Grains

**DOI:** 10.1371/journal.pone.0148712

**Published:** 2016-02-09

**Authors:** Gangcheng Wu, Stuart K. Johnson, Janet F. Bornman, Sarita J. Bennett, Vijaya Singh, Azra Simic, Zhongxiang Fang

**Affiliations:** 1 Food Science and Technology Program, School of Public Health, Curtin University, Perth, WA, 6845, Australia; 2 International Institute of Agri-Food Security (IIAFS), Curtin University, PO Box U1987, Perth, WA, 6845, Australia; 3 Department of Environment and Agriculture, School of Science, Curtin University, Perth, WA, 6845, Australia; 4 Queensland Alliance for Agriculture and Food Innovation, The University of Queensland, Brisbane, Qld, 4072, Australia; 5 National Measurement Institute, Perth, WA, 6151, Australia; 6 Faculty of Veterinary and Agricultural Sciences, The University of Melbourne, Parkville, Victoria, 3010, Australia; Aberystwyth University, UNITED KINGDOM

## Abstract

**Background:**

It has been predicted that the global temperature will rise in the future, which means crops including sorghum will likely be grown under higher temperatures, and consequently may affect the nutritional properties.

**Methods:**

The effects of two growth temperatures (OT, day/night 32/21°C; HT 38/21°C) on tannin, phytate, mineral, and *in vitro* iron availability of raw and cooked grains (as porridge) of six sorghum genotypes were investigated.

**Results:**

Tannin content significantly decreased across all sorghum genotypes under high growth temperature (*P* ≤0.05), while the phytate and mineral contents maintained the same level, increased or decreased significantly, depending on the genotype. The *in vitro* iron availability in most sorghum genotypes was also significantly reduced under high temperature, except for Ai4, which showed a pronounced increase (*P* ≤0.05). The cooking process significantly reduced tannin content in all sorghum genotypes (*P* ≤0.05), while the phytate content and *in vitro* iron availability were not significantly affected.

**Conclusions:**

This research provides some new information on sorghum grain nutritional properties when grown under predicted future higher temperatures, which could be important for humans where sorghum grains are consumed as staple food.

## Introduction

Sorghum (*Sorghum bicolor* (L.) Moench) is the fifth leading cereal crop in the world after rice, wheat, barley and maize. It provides a staple food for people living in resource-poor households in many parts of the developing world, particularly in semi-arid areas such as sub-Saharan Africa [1]. Various foods are made from sorghum grain, with porridge being one of the most important. However, the presence of anti-nutritional factors, such as phytate and tannins, has significant negative effects on the nutritional quality of sorghum. For example, both phytate and tannins can bind to minerals, such as iron (Fe), and reduce its digestibility and bioavailability to cause iron deficiency in humans [2–4]. Low iron bioavailability may cause some physical problems such as tiredness and shortness of breath [5].

Phytate and tannin contents in sorghum grains vary with genotype [6]. Sorghum genotypes with the *B1-B2-* gene contain high levels of tannin, and tannin levels can vary about two fold between genotypes which can have important consequences for the bioavailability of iron in the grain [3, 7]. However, little information is available on tannin, phytate and mineral variability in the sorghum genotypes from the Australian sorghum breeding program, and whether the levels of these compounds are influenced by temperature during plant growth.

Temperature is an important factor controlling the growth and development of the sorghum plant. According to published climate models, the global mean surface temperature will rise by 1.8 to 4.0°C by the year 2100, which means crops including sorghum will likely be grown under higher temperatures in the future [8]. Only very limited research has been reported to date on the effect of temperature during plant growth on the chemical composition of sorghum grain. For example, the free phenolic compounds in white pericarp sorghum increased under a warm and moist environment [9]. However, well controlled studies on the effect of genotype and temperature and their interaction during plant growth on sorghum grain production and composition are limited. Li et al. [10] and Nguyen et al. [11] planted diverse sorghum genotypes in temperature-controlled growth chambers and found seed-set of all genotypes, plus the activities of starch biosynthetic enzymes were reduced under a high temperature regime. To date, no published information is available on the effects of genotype and temperature during plant growth on sorghum grain tannin, phytate and mineral levels, or the associated iron availability.

The objective of this study was to determine the effects of two different temperature regimes during plant production on grain phytate, tannin and minerals levels and *in vitro* iron availability in six different sorghum genotypes from the Australian sorghum breeding program.

## Materials and Methods

### Grain growth experimental details

Six sorghum genotypes with three different pericarp colours (red, brown and white) and two breeding lines (hybrid and inbred) were used in the study (Table 1). CHH1, CHH2 and AQL33/QL36 all originated from Australia. Ai4, PI563516 and IS8525 originated from China, Mali and Ethiopia, respectively. They were supplied by the Australia sorghum pre-breeding program which is a partnership between the University of Queensland, The Queensland Department of Agriculture and Fisheries and the Grains research and Development Corporation. The sorghum cultivars were grown in the Controlled Environment Facility, Queensland Bioscience Precinct, University of Queensland, St Lucia, Brisbane, Australia in 2013. Two growth chambers were used to grow three replicate plants of each genotype under two different temperature regimens: optimum temperature (OT) of 32°C maximum, 21°C minimum; and high temperature (HT) of 38°C maximum, 21°C minimum. The experimental details of the temperature growth trial within the Controlled Environment Facility have been reported in detail previously [11]. After harvest, the grain was air-dried to 10% moisture content and manually cleaned after which it was vacuum-packed and kept at 4°C in the dark until analysis.

**Table 1:** Breeding line and grain pericarp/testa colour of sorghum genotypes used for temperature trials.

Sample number	Genotype	Inbred line/hybrid	Grain pericarp/testa colour
1	CHH2	Commercial hybrid	Red
2	CHH1	Commercial hybrid	Red
3	AQL33/QL36	Hybrid	Red
4	Ai4	Inbred	Red
5	PI563516	Inbred	White
6	IS8525	Inbred	Brown

### Sample preparation

#### Flour

Sorghum whole grain samples were ground for 5 min using a grain mill CEMOTEC 1090 (Foss Tecator, Höganäs, Sweden), to enable 100% of the flour to pass through a 500 μm sieve. The flour was vacuum-packed and stored at 4°C in the dark until analysis and porridge preparation.

#### Porridge

The sorghum whole grain flours were made into a thick porridge with three replications by the method described by Kruger, Taylor and Oelofse [7]. The porridge was freeze-dried with a rotational vacuum concentrator 2–18 CDplus freeze drier (Christ, Osterote, Lower Saxony, Germany) at -30°C and 0.37 mbar for 20 h. The dried porridge samples were passed 100% through a 500 μm sieve to break down agglomerates before analysis. Ground samples were vacuum packed and stored at 4°C until analysis. Each sample was prepared in triplicate

### Analytical methods

#### Condensed tannin content

Condensed tannin content of the raw flours and dried porridge was determined by the vanillin HCl assay as described by Price, Van Scoyoc and Butler [12]. Ten ml acidified methanol (1% HCl in methanol) was added to 1 g of sorghum flour or dried porridge in triplicate, and the mixture was extracted for 20 min with constant shaking at room temperature. The extracted sample was centrifuged at 3,220 g for 10 min at room temperature and the supernatant collected for analysis. The residue was re-extracted twice with 5 ml acidified methanol. Vanillin HCl reagent (equal volumes of 2% vanillin in methanol and 8% concentrated HCL in methanol) was prepared daily. To 1 ml of extract was added 5 ml of vanillin HCl reagent. The solution was mixed and incubated for 20 min at room temperature. The absorption of the resulting solution was measured at 500 nm on a UV-1800 spectrophotometer (Shimadzu, Canby, USA). Catechin was used as the standard for the calibration curve. Condensed tannin content was expressed as g catechin equivalents (CE) Kg^-1^ sample (dry basis (db)).

#### Phytate content

Phytate content of the raw flours and dried porridge was measured using the spectrophotometric method of Haug and Lantzsch [13]. In brief, the sample solution of ground sorghum (about 0.5 g) was extracted from both raw flour and dried porridge with 20 ml of 0.2 M HCl for 90 min in a shaking water bath at 25°C. The solution was centrifuged at 3,220 g for 10 min after extraction at room temperature, and the supernatant collected. The residue was washed twice with 10 ml 0.2 M HCl, centrifugation was repeated between each washing and the resulting supernatants combined. The combined supernatants were made up to 50 ml with 0.2 M HCl. Each sample was extracted in duplicate.

An 0.5 ml aliquot of the supernatant or a standard phytate solution (phytate phosphorus concentrations from 3–30 μg/ml) was pipetted into capped test tubes. A 2 ml ferric solution (0.2 g FeNH_4_(SO_4_)_2_ 12 H_2_O in 1L 0.2 M HCl) was added to each tube and mixed thoroughly. The capped tubes were heated in a boiling water bath for 30 min, after which they were cooled in ice water to room temperature. Two ml of 2,2′-bipyridyl solution (10 g 2,2′-bipyridyl and 10 ml thioglycollic acid in 1 L distilled water) was added. After mixing thoroughly, the absorption of the solutions was measured at 519 nm on a UV-1800 spectrophotometer (Shimadzu, Canby, USA). Absorption must be read within 0.5–1 min because reactions between bipyridine and iron phytate can change the colour with time. Distilled water was used as a blank. Phytate phosphorus was used as the standard. Phytate content was expressed as g Kg^-1^ sample (db).

#### Mineral analysis

Mineral content of the raw flours was measured using a Vista Pro inductively coupled plasma optical emission spectrometer (ICP-OES) (Varian, Palo Alto, USA) at the National Measurement Institute, Perth, Western Australia. Sorghum flour (1 g) was digested with 3 mL of concentrated HNO_3_ plus 3 mL of concentrated HCl in a DEENA automated digestion block (Thomas Cain, Nebraska, USA) at 95°C for 2 hours.

After digestion, the sample tubes were made up to 40 ml with distilled water, and solutions were left to settle. Samples were diluted 5-fold with distilled water before they were analysed using the ICP-OES. Appropriate emission wavelengths that had higher sensitivity and lower interferences were chosen to analyse elements of Fe, Ca, P and Zn. The wavelengths used to measure iron, calcium, phosphorus and zinc were 238.204nm, 327.395nm, 117.434nm and 213.857nm, respectively. All results were expressed as g Kg-1 sample (db).

#### In *vitro iron* availability

The dialysis method as described by Luten et al [14] was used to estimate the *in vitro* iron availability of raw flour and dried porridge samples. Two equal amounts of samples (around 1 g) were weighted, and each sample was mixed with 5 ml distilled water, and the pH adjusted to 2 with 6 M HCl. For simulated gastric digestion, 0.15 ml of freshly prepared pepsin solution (4 g pepsin, 2,500 units/mg, from Toxfree (Bedford St, Gillman SA)) dissolved in 25 ml 0.1 M HCl was added to each sample and the mixture incubated at 37°C for 2 h in a shaking water bath. Titratable acidity was determined in one sample of the gastric digest in which a freshly prepared pancreatin-bile extract mixture (400 mg pancreatin from Toxfree (Gillman, SA, Australia) and 2.5 g bovine bile extract from Sigma (St. Louis, MO, USA) in 100 ml 0.1 M NaHCO_3_) was added, and the pH adjusted to 7.5 with 0.2 M NaOH. Titratable acidity of the samples was defined as the amount of 0.2 M NaOH required to reach the pH 7.5.

In the simulated intestinal stage, segments of dialysis tubing with a molecular mass cut off at 10 kDa (Thermo Scientific, IL, USA) containing 25 ml of sodium bicarbonate (equivalent in moles to the NaOH used to measure the titratable acidity), were placed in tubers with the gastric digest and incubated at 37°C for 30 min, after which 5 ml pancreatin-bile solution were added into tubers and incubated for a further 2 h. The iron content in the dialysis bag was determined as described in Section 2.3.4.

### Statistical analysis

The analysis of tannin, phytate, minerals and *in vitro* iron availability were repeated in triplicate and the results were expressed as the mean ± standard deviation (SD). The data were statistically analysed using SPSS V20 (SPSS Inc., an IBM Company, Chicago, IL, USA). The main effects of genotype (G) and temperature (T) and their interaction were investigated by two-way ANOVA. Tukey post hoc tests were used to separate individual sample means. Pearson correlation was used to investigate the associations between in vitro iron availability and each of condensed tannin and phytate levels. *P* ≤ 0.05 indicated significant difference.

## Results and Discussion

### Grain physical characteristics and seed set

The pollen germination and seed-set percentage, which are positively related to sorghum yields, of all genotypes under both temperature treatments have been reported previously in detail [11]. HT significantly reduced pollen germination and seed-set percentage of all genotypes, and genotypes differed significantly in pollen viability percentage (17–63%) and seed-set percentage (7–65%) under HT [11], indicating the sorghum production yields were reduced by high temperature, the extent depending on the genotypes. The physical characteristics including hardness, weight and diameter of different sorghum genotype grains with two temperature treatments were recorded [S1 Dataset]. Apart from CHH1, HT increased the grain weight and diameter of all other sorghum genotypes, indicating that most genotypes might be suitable to grow under predicted future higher temperatures. The seed set of these samples were also measured and reported in the work of Nguyen et al. [11], which showed that these selected genotypes have relatively high percentage of seed set under HT, compared to other genotypes. Singh et al. [15] demonstrated that sorghum seed set percentage had a positive correlation with grain yield. Therefore, the sorghum genotypes used in this study might have good tolerance to increased temperature compared to other genotypes in terms of yield.

### Tannin

The influence of genotype and temperature during plant growth on the grain tannin content is presented in Table 2. Temperature, genotype and their interaction had a significant effect on tannin content of the uncooked whole grains (*P* ≤0.05). There were wide variations in the tannin content ranging from 2.16 to 42.01 g CE Kg^-1^, with genotype of AQL33/QL36 and CHH1 having the lowest (*P* ≤0.05) and IS8525 having the highest (*P* ≤0.05) content, respectively, under OT. However, under HT, the tannin levels in the six sorghum whole grain genotypes were significantly lower than under OT and ranged from 0.79 to 32.42 g CE Kg^-1^, with PI563516 and IS8525 having the lowest and highest levels (*P* ≤0.05), respectively (Table 2). This implies that tannin biosynthesis in the sorghum grains might be inhibited under the higher growing temperature in the study. It has been reported that tannin is polymerized from other polyphenols (catechin and epicatechin), but the mechanism still remains speculative [16, 17].

**Table 2:** Influence of genotype and temperature during plant growth under optimal temperature (OT) and high temperature (HT) on tannin content (g catechin equivalents Kg^-1^ db) in sorghum whole grain flour (uncooked) and porridge.

Genotype	Whole grain flour (OT)	Whole grain flour (HT)	Porridge (OT)	Porridge (HT)
CHH2	2.73±0.11 ^b^^C^	1.49±0.07 ^c^^B^	1.91±0.12 ^d^^B^	0.34±0.04 ^a^^A^
CHH1	2.29±0.03 ^a^^D^	1.67±0.06 ^c^^C^	1.14±0.18 ^b^^B^	0.68±0.05 ^a^^A^
AQL33/QL36	2.16±0.09 ^a^^D^	1.00±0.05 ^b^^C^	0.84±0.05 ^a^^B^	0.49±0.02 ^a^^A^
Ai4	4.13±0.13 ^c^^D^	3.40±0.27 ^d^^C^	1.46±0.04 ^c^^B^	0.38±0.03 ^a^^A^
PI563516	3.02±0.43 ^b^^C^	0.79±0.03 ^a^^B^	0.72±0.05 ^a^^B^	0.46±0.07 ^a^^A^
IS8525	42.01±2.22 ^d^^D^	32.42±0.66 ^e^^C^	5.08±0.07 ^e^^B^	4.02±0.03 ^b^^A^

^a, b, c, d, e^ Values with different letters in the same column are significantly different (*P* ≤0.05).

^A, B,C, D^ Values with different letters in the same row are significantly different (*P* ≤0.05)

It has previously been reported that sorghum genotypes with a pigmented testa contained significantly higher levels of tannin than those without a pigmented testa [18]. In the present study, the tannin content of IS8525 was about 10–15 times higher than the others, suggesting that IS8525 may have both dominant *B*_*1*_ and *B*_*2*_ genes, which control the presence or absence of a pigmented testa. Interestingly, although high temperature could significantly reduce the tannin content in all genotypes, the tannin content was still mainly depended on genotype (Table 2).

Sorghum grains are usually cooked prior to human consumption, so understanding changes in tannins during the cooking process is important. The results indicate that cooking into a thick porridge significantly reduced the tannin content in all the sorghum genotypes (*P* ≤0.05), irrespective of the growing temperature (Table 2). The tannin content in the sorghum porridge ranged from 0.72 to 5.08 g CE Kg^-1^ under OT, and from 0.38 to 4.02 g CE Kg^-1^ under HT. The tannin contents in the porridge were still the highest in the genotype IS8525, irrespective of growing temperature, and were the lowest in PI563516 and CHH2 (*P* ≤0.05) for the OT, whereas at HT porridges of all of the genotypes except IS8525 had similar levels (*P* >0.05). After cooking, there was a large reduction in tannin content (about 87%) in IS8525, while in the other five genotypes it was reduced by between 30 and 76%. These results are in agreement with those of Dlamini, Taylor and Rooney [19] and Wedad et al. [20], who showed that tannin content in sorghum grain was reduced through cooking by 36%–80%. This might be the phenolic hydroxyl groups of tannin reacting with food compounds (protein and minerals such as iron) to form insoluble complexes during the heating process [21]. Since the measurable tannin content was lower after cooking, this may help to reduce the undesirable astringent and bitter taste caused by tannins in the grain.

However, tannin has been commonly referred to as an anti-nutritional factor, because of its high affinity to proteins and tendency to complex with divalent metal ions, thus decreasing their availability [22, 23]. However, these negative effects can be reduced or eliminated by decreasing the tannin content in sorghum grains through cooking. In the present experiment, the tannin content was also significantly reduced by growing under the higher temperatures (Table 2). This suggests that sorghum could contain lower levels of tannin under the expected higher future temperatures, which would increase its nutritional value as human food or animal feed. Tannin has also been identified as the major contributor of the antioxidant activity of some genotypes of sorghum grains and tannins may have gastro-protective and cholesterol-lowering properties [24]. For future production of high tannin sorghum for manufacture into high antioxidant foods, the genotype IS8525 could be a good candidate since it contains by far the highest levels of tannin in both OT and HT conditions in the present study (Table 2).

### Phytate content

The phytate content of the whole grain flours among the six sorghum genotypes varied between 10.60 g Kg^-1^ (CHH2) and 17.44 (IS8525) g Kg^-1^ under OT (Table 3). There was a significant effect of genotype, temperature and their interaction on phytate content in the uncooked flours (*P* ≤0.05). The phytate values reported here are in the range previously reported for sorghum (2.11–17.62 g Kg^-1^) [20]. HT had diverse impacts on phytate of sorghum grain. For three hybrid lines with the same red colour (Table 1), the phytate in CHH1 and CHH2 increased 32.24% and 26.13% respectively but showed no significant changes in AQL33/QL36 when growth temperature was increased. The white inbred line: PI563516 had higher levels of phytate while the other two (brown and white) showed no significant changes under HT. These results suggest that the phytate content in sorghum grain was not affected or increased by HT, depending on the genotype. The effect of temperature during plant growth on the phytate content in sorghum grains has not been reported previously. In an experiment on other crops, including maize, however, Horvatic and Balint [25] found that lower temperatures and increased irrigation decreased the phytate levels, but they did not separate the influence of temperature and irrigation.

**Table 3:** Influence of genotype and temperature during plant growth under optimal temperature (OT) and high temperature (HT) on the phytate content (g Kg^-1^, db) of sorghum whole grain flour (uncooked) and porridge.

Genotype	Whole grain flour (OT)	Whole grain flour (HT)	Porridge (OT)	Porridge (HT)
CHH2	10.60±0.39 ^a^^A^	13.73±0.13 ^a^^B^	10.61±1.13 ^a^^A^	12.98±0.50 ^a^^B^
CHH1	12.25±0.49 ^b^^A^	16.20±0.74 ^c^^B^	12.37±0.15 ^b^^A^	16.74±0.47 ^c^^B^
AQL33/QL36	15.80±0.23 ^d^^A^	15.02±0.82 ^b^^A^	14.89±0.21 ^d^^A^	14.37±0.17 ^b^^A^
Ai4	15.56±0.23 ^d^^A^	15.92±0.82 ^b^^c^^A^	15.53±0.52 ^d^^A^	15.70±1.62 ^b^^c^^A^
PI563516	14.07±0.10 ^c^^B^	12.96±0.94 ^a^^A^	13.68±0.92 ^c^^B^	12.87±0.63 ^a^^A^
IS8525	17.44±0.40 ^e^^A^	16.39±0.41 ^c^^A^	16.47±0.89 ^e^^A^	16.24±0.13 ^c^^A^

^a, b, c, d, e^ Values with different letters in the same column are significantly different (*P* ≤0.05).

^A, B^ Values with different letters in the same row are significantly different (*P* ≤0.05).

The phytate level in the porridge was not significantly different to that in the raw whole grain flour for all sorghum genotypes (*P* > 0.05) within each growth temperature (Table 3). Endogenous phytases that are present in sorghum grains have the potential to break down phytate. However, they may have been deactivated by heat during the porridge preparation [26]. Previous research found that phytate in sorghum seeds was reduced under the traditional porridge preparation method, probably because of longer cooking time [20] than that used in our research. In the present study, cooking into a simple porridge did not reduce the phytate content. Phytate chelates metal ions, such as iron, to form insoluble complexes in the gastrointestinal tract, and these irons cannot be digested [27]. These results might indicate that cooking process cannot improve the mineral bioavailability.

### Mineral content

The iron content of the whole grain flour, which ranged from 14.07 to 47.83 mg/kg with PI563516 having the lowest and CHH1 having the highest level (*P* ≤0.05) under OT (Table 4), was significantly affected by genotype and temperature (*P* ≤0.05). It is interesting to note that the iron content in the hybrid lines (36.9×10^−3^–47.83×10^−3^ g kg^-1^) was significantly higher than that in the inbred lines (14.07×10^−3^–37.43×10^−3^ g kg^-1^) (*P* ≤0.05). A significant difference has previously been reported between the sorghum genotypes in iron acquisition and storage during plant growth and in particular among breeding lines [7]. Under HT the iron content in the whole grain flours varied between 19.57×10^−3^ and 54.53×10^−3^ g kg^-1^, with the level in CHH1 and PI563516 remaining the lowest and highest respectively (*P* ≤0.05) (Table 4). Temperature was found to have a marked effect on the iron content in some of the sorghum flours (*P* ≤0.05). For example, the iron content in CHH1 and IS8525 was significantly higher (*P* ≤0.05) in HT than OT, whereas that of AQL33/QL36 was lower under HT (*P* ≤0.05).

**Table 4:** Influence of genotype and growing temperature during plant growth under optimal temperature (OT) and high temperature (HT) on mineral (Fe, Ca, P, Zn) contents (g kg^-1^ db) in sorghum wholegrain flour (uncooked).

Genotype	Fe		Ca		P		Zn	
	OT	HT	OT	HT	OT	HT	OT	HT
CHH2	36.90×10^−3^±0.10×10^-3^^c^^B^	33.47×10^−3^±0.15×10^−3^ ^b^^A^	99.47×10^−3^±0.55×10^−3^ ^e^^A^	105.33×10^−3^±2.51×10^−3^ ^d^^B^	3.156±0.055 ^a^^A^	3.636±0.038 ^b^^B^	20.17×10^−3^±0.21×10^−3^ ^b^^A^	21.06×10^−3^±1.00×10^−3^ ^c^^A^
CHH1	47.83×10^−3^±0.47×10^−3^ ^e^^A^	54.53×10^−3^±1.16×10^−3^ ^e^^B^	66.00×10^−3^±3.21×10^−3^ ^c^^A^	85.37×10^−3^±3.57×10^−3^ ^c^^B^	3.460±0.025 ^b^^A^	4.490±0.036 ^d^^B^	20.70×10^−3^±0.35×10^−3^ ^b^^A^	24.57×10^−3^±0.75×10^−3^ ^d^^B^
AQL33/QL36	45.37×10^−3^±0.38×10^−3^ ^d^^B^	35.57×10^−3^±1.21×10^-3^^c^^A^	88.57×10^−3^±2.37×10^−3^ ^d^^B^	63.26×10^−3^±0.53×10^−3^ ^a^^A^	4.215±0.013 ^c^^B^	3.957±0.031 ^c^^A^	19.30×10^−3^±0.17×10^−3^ ^b^^B^	17.50×10^−3^±0.46×10^−3^ ^b^^A^
Ai4	37.43×10^−3^±0.75×10^−3^ ^c^^A^	35.57×10^−3^±0.31×10^−3^ ^c^^A^	96.47×10^−3^±1.32×10^−3^ ^e^^B^	87.17×10^−3^±1.92×10^−3^ ^c^^A^	3.480±0.010 ^b^^A^	3.680±0.043 ^b^^B^	27.03×10^−3^±0.84×10^−3^ ^d^^A^	26.80×10^−3^±0.63×10^−3^ ^e^^A^
PI563516	14.07×10^−3^±0.15×10^−3^ ^a^^A^	19.57×10^−3^±0.31×10^−3^ ^a^^B^	50.87×10^−3^±0.77×10^−3^ ^a^^A^	65.74×10^−3^±0.15×10^−3^ ^a^^B^	3.200±0.036 ^a^^B^	2.980±0.075 ^a^^A^	12.33×10^−3^±0.15×10^−3^ ^a^^A^	16.10×10^−3^±0.20×10^−3^ ^a^^B^
IS8525	30.53×10^−3^±0.25×10^−3^ ^b^^A^	40.37×10^−3^±0.66×10^−3^ ^d^^B^	58.40×10^−3^±0.26×10^−3^ ^b^^A^	70.30×10^−3^±1.18×10^−3^ ^b^^B^	3.963±0.051^b^^A^	3.593±0.015 ^c^^B^	24.40×10^−3^±0.26×10^−3^ ^c^^A^	30.63×10^−3^±0.06×10^−3^ ^f^^B^

^a, b, c, d, e, f^ Values with different letters in the same column are significantly different (*P* ≤0.05).

^A, B^ Values with different letters in the same genotype and the same mineral are significantly different (*P* ≤0.05).

The calcium content of the sorghum is also shown in Table 4, and was significantly affected by genotype and temperature (*P* ≤0.05). The six sorghum genotypes varied significantly (*P* ≤0.05) in their calcium content, ranging from 50.87×10^−3^ to 99.47×10^−3^ g kg^-1^ flour under OT and 63.26×10^−3^ to 105.33×10^−3^ g kg^-1^ flour under HT. The calcium levels in only two of the sorghum genotypes (AQL33/QL36 and Ai4) were significantly reduced under high growing temperatures, while in the others they were significantly increased (*P* ≤0.05), also suggesting a genotype influence.

Phosphorus is the major mineral in sorghum and its content in the sorghum grains was significantly influenced by the genotypes and temperature (*P* ≤0.05). A 1.015 g kg^-1^ flour difference was recorded between AQL/QL36 (the highest level) and PI563516 (the lowest level) under OT. An increase in day temperature from 32 to 38°C resulted in a notable decrease in the phosphorus content in the grains of AQL33/QL36 and PI563516 (*P* ≤0.05).

Zinc content was significantly affected by genotype and temperature (*P* ≤0.05), and ranged from 12.33×10^−3^to 27.03×10^−3^ g kg^-1^ flour under OT, and from 16.10×10^−3^ to 26.80×10^−3^ g kg^-1^ flour under HT, respectively (Table 4). The zinc content in other sorghum varieties has been reported between 14×10^−3^ and 38×10^−3^ g kg^-1^ [28] Zinc levels were unchanged in Ai4 and CHH2, decreased in AQL33/QL36 (*P* ≤0.05), but increased in the other 3 genotypes (*P* ≤0.05) when the growing temperature was increased from 32/21°C to 38/21°C.

As sorghum is one of the staple cereal grains in Africa, sorghum grains may provide the major source of minerals for the local people. In this research, the three sorghum hybrid lines of CHH2 and CHH1with high mineral content are likely suitable genotypes to be grown in a high temperature environment as a good dietary mineral source.

### In *vitro iron* availability

The *in vitro* iron dialysability of the whole grain raw flours (as an estimate of availability) is shown in Table 5. Genotype, temperature and their interaction had a significant effect on the *in vitro* iron availability (*P* ≤0.05) of the uncooked whole grain flours. Under OT the significantly highest *in vitro* iron availability (*P* ≤0.05) was found in CHH2, while the lowest (*P* ≤0.05) occurred in IS8525. Values varied from 0.87 to 5.94% for the genotypes grown under OT but ranged from 0.47 to 3.15% under HT, indicating the *in vitro* iron dialysability in most tested sorghum genotypes was decreased, with the exception of genotype Ai4 which was significantly increased, irrespective of tannins and phytate content (Tables 2 and 3). Interestingly, the flour from IS8525 had the lowest levels of *in vitro* iron availability under both growing temperatures. This may be related to the fact that IS8525 had the extremely high levels of tannin, which might exert a significant inhibitory effect on iron absorption [29]. One of the postulated mechanisms of the action of tannins is chelation of pro-oxidant metals like iron [30].

**Table 5:** Influence of genotype and temperature during plant growth under OT and HT on *in vitro* iron dialysability (%) of sorghum whole grain flour and porridge.

Genotype	Whole grain flour (OT)	Whole grain flour (HT)	Thick porridge (OT)	Thick porridge (HT)
CHH2	5.94±0.13 ^f^^B^	1.79±0.09 ^c^^A^	5.76±0.45 ^f^^B^	1.67±0.10 ^c^^A^
CHH1	4.70±0.07 ^e^^B^	3.15±0.17 ^d^^A^	4.54±0.15 ^e^^B^	3.21±0.11 ^d^^A^
AQL33/QL36	1.54±0.11 ^b^^B^	1.10±0.24 ^b^^A^	1.47±0.12 ^b^^B^	0.89±0.04 ^b^^A^
Ai4	2.61±0.06 ^c^^A^	3.10±0.08 ^d^^B^	2.42±0.23 ^c^^A^	3.18±0.23 ^d^^B^
PI563516	3.09±0.21 ^d^^B^	1.07±0.15 ^b^^A^	2.92±0.13 ^d^^B^	0.95±0.12 ^b^^A^
IS8525	0.87±0.09 ^a^^B^	0.47±0.08 ^a^^A^	0.83±0.06 ^a^^B^	0.44±0.05 ^a^^A^

^a, b, c, d, e, f^ Values with different letters in the same temperature column are significantly different (*P* ≤0.05).

^A, B^ Values with different letters in the same row are significantly different (*P* ≤0.05).

The *in vitro* iron availability in the porridge varied between 0.83 and 5.76% for the OT samples and between 0.44 and 3.21% for the HT samples (*P* ≤0.05). The cooking process had no significant effect on the iron dialysability compared to the raw whole grain flours (*P* > 0.05). This could partly be due to the phytate not being affected by the heat treatment (section 3.2), as phytate can chelate irons to form insoluble complexes to decrease iron dialysability. Kruger et al. [7] observed similar results when they prepared thick porridge using four different sorghum genotypes. However, some researchers reported that heat treatment could slightly increase [28] or decrease [31] the *in vitro* iron availability. The *in vitro* iron availability is strongly affected by the levels of polyphenols and phytate, and Towo et al. [2] also proposed that the reduction of the phenolic content without the reduction of phytate would not improve the iron availability. The possible reason was that although cooking can reduce the total measurable tannin content, the amount of tannin bound with iron may have increased during cooking [7]. Some researchers have investigated the effect of reducing phytate in tannin and tannin-free sorghum varieties by fermentation, and reported that only tannin-free sorghum varieties increased their iron availability after phytate reduction [20]. Their results suggested that the *in vitro* iron availability in sorghum grains could be increased by reducing both phytate and tannin content. In this experiment, cooking could significantly decrease tannin content but had no effects on the phytate content, so the iron dialysability was not improved after cooking.

Levels of *in vitro* iron availability in sorghum grains was significantly negatively correlated with tannin (Pearson’s correlation: r = 0.463, *P* ≤0.05) and phytate (r = 0.694, *P* ≤0.05) content. Other researchers have also confirmed that a significant negative correlation was observed between iron availability and the tannin and phytate content [32, 33].

From the present study, production under HT gave reduced *in vitro* iron dialysability of the raw whole grain and porridge of some sorghum genotypes, so the nutritional values of these foods, from an iron availability view, could decrease under predicted higher temperatures in the future. In terms of sorghum porridge as a staple food for people living in malnourished communities in rural Africa, the sorghum genotype CHH2, which had higher *in vitro* iron availability than the other sorghum genotypes, could be a suitable available iron resource. However, under high temperature production conditions, the sorghum genotypes CHH1 and Ai4 have potential for high *in vitro* iron availability.

## Conclusions

Analysis of the tannin, phytate, mineral (Fe, Ca, P, Zn) contents and *in vitro* iron dialysability of whole grain flours and porridge of six diverse sorghum genotypes of varied grain colours grown under OT and HT provided new evidence of effects of both genotype and temperature of plant growth. Genotype had a significant effect on tannin, phytate, mineral contents and *in vitro* iron dialysability of raw grain flours. The tannin content in all investigated sorghum grains decreased under the higher growth temperature. However, the high growth temperatures had diverse effects on the phytate, mineral contents and *in vitro* iron availability of the sorghum grains, depending on the genotype. This study has the potential to help farmers to select appropriate sorghum genotypes to grow for maximum iron bio-avaiability at elevated production temperatures that may occur in the future. However, more research is required to discover the plant physiological mechanisms behind the changes in tannin, phytate and *in vitro* iron availability under high growth temperatures.

## Supporting Information

S1 Dataset S1 Dataset contains data on grain physical characteristics of six sorghum genotypes under optimal temperature (OT) and high temperature (HT). (XLSX)

S2 Dataset S2 Dataset contains data on phytate, tannin and *in vitro* iron availability in six sorghum whole grain flour (uncooked) and porridge under optimal temperature (OT) and high temperature (HT). (XLSX)

S3 Dataset S3 Dataset contains data on mineral (Fe, Ca, P, Zn) contents of six sorghum genotypes under optimal temperature (OT) and high temperature (HT). (XLSX)
